# First aid strategies that are helpful to young people developing a mental disorder: beliefs of health professionals compared to young people and parents

**DOI:** 10.1186/1471-244X-8-42

**Published:** 2008-06-08

**Authors:** Anthony F Jorm, Amy J Morgan, Annemarie Wright

**Affiliations:** 1 ORYGEN Research Centre, Department of Psychiatry, University of Melbourne, Locked Bag 10, Parkville, Victoria, Australia

## Abstract

**Background:**

Little is known about the best ways for a member of the public to respond when someone in their social network develops a mental disorder. Controlled trials are not feasible in this area, so expert consensus may be the best guide.

**Methods:**

To assess expert views, postal surveys were carried out with Australian GPs, psychiatrists and psychologists listed on professional registers and with mental health nurses who were members of a professional college. These professionals were asked to rate the helpfulness of 10 potential first aid strategies for young people with one of four disorders: depression, depression with alcohol misuse, social phobia and psychosis. Data were obtained from 470 GPs, 591 psychiatrists, 736 psychologists and 522 mental health nurses, with respective response rates of 24%, 35%, 40% and 32%. Data on public views were available from an earlier telephone survey of 3746 Australian youth aged 12–25 years and 2005 of their parents, which included questions about the same strategies.

**Results:**

A clear majority across the four professions believed in the helpfulness of listening to the person, suggesting professional help-seeking, making an appointment for the person to see a GP and asking about suicidal feelings. There was also a clear majority believing in the harmfulness of ignoring the person, suggesting use of alcohol to cope, and talking to them firmly. Compared to health professionals, young people and their parents were less likely to believe that asking about suicidal feelings would be helpful and more likely to believe it would be harmful. They were also less likely to believe that talking to the person firmly would be harmful.

**Conclusion:**

Several first aid strategies can be recommended to the public based on agreement of clinicians about their likely helpfulness. In particular, there needs to be greater public awareness of the helpfulness of asking a young person with a mental health problem about suicidal feelings.

## Background

Because of the high prevalence of mental disorders in the community, every person will either develop a disorder themselves or have close contact with someone who does. For this reason, it has been argued that members of the public need some degree of knowledge about the recognition, management and prevention of these disorders – what has been termed "mental health literacy" [1]. However, surveys in a number of countries have found deficits in public knowledge, including inability to recognize mental disorders, negative views about some standard psychiatric treatments, particularly medications, and positive views about some non-evidence-based interventions [2-8].

An important aspect of mental health literacy, which has received comparatively little attention, is the initial response of those in the social network when someone is developing a mental disorder. When a person develops a mental disorder, they will often not receive any professional help or there may be delays before they get this help [9]. Nevertheless, the changes in the person's behaviour and functioning are likely to be apparent to family, friends and others in their social network. Indeed, family and friends are seen as important sources of help for mental disorders by both adults and adolescents [8,10-12]. These people are in a position to provide first aid to the person with the mental disorder and to facilitate professional help-seeking. *Mental health first aid *can be defined as the help provided to a person developing a mental health problem or in a mental health crisis. The first aid is given until appropriate professional treatment is received or until the crisis resolves.

A number of community surveys have examined the mental health first aid skills of the public. In a national survey of Australian adults, Jorm et al. [13] presented participants with a vignette of a person with a mental disorder and asked what they would do if that person was someone they had known for a long time and cared about. The responses to this open-ended question were coded into categories. The most common responses were to encourage professional help-seeking and to listen to and support the person, although significant minorities did not give these responses. Other first aid responses were mentioned by only a minority. Of particular concern was the low percentage assessing the risk of harm for a vignette which portrayed a depressed person with suicidal thoughts.

More recently, Jorm et al. [14] have reported a similar survey of young Australians aged 12–25 years and their parents. The young people were presented with a vignette and asked how they would respond to a peer with the problem. Parents were given the same vignettes and asked how they would respond if this was their child. Only a minority of young people mentioned that they would encourage professional help, even when the vignette portrayed a psychotic person. However, most parents said they would encourage professional help-seeking, although there was a minority who did not mention it. Only a minority of both young people and parents mentioned listening to the person's problems and few mentioned asking about suicidal thoughts.

In a survey of high school students, Kelly et al. [15] asked how they would respond to a peer portrayed in a vignette of either depression or conduct disorder. Around half the sample gave positive social support as their response, but only a minority would engage the help of an adult such as a parent, teacher or school counsellor. Other research has examined how young adults would respond to a suicidal peer and found that many would not tell a responsible adult about it [16].

While these surveys indicate that the public's first aid skills may not be optimal, there is a difficulty judging the adequacy of their responses, because there is no evidence-base or guidelines on what is appropriate first aid for mental disorders. It is not feasible to carry out controlled trials on first aid responses of the public. In this situation, the best guide is probably expert consensus. Here we report the findings from surveys of a range of health professionals on the likely helpfulness or harmfulness of 10 first aid strategies for young people. These strategies were rated for vignettes depicting four disorders: depression, depression with alcohol misuse, social phobia and psychosis. The vignettes focussed on young people because mental disorders often have first onset during youth [17]. Furthermore, young people show a strong preference for getting initial help from their family and friends [11,18] who are therefore a potential source of first aid. Because health professionals vary in the sorts of disorders they have experience with and in the interventions they are trained to use, we sought the views of a range of professions (GPs, psychiatrists, psychologists and mental health nurses) and looked for consensus across these groups. The consensus views of these professionals can then give guidance on what the public should be advised to do. The questions used in these surveys were also included in an earlier national survey of Australian youth and their parents [14], allowing an examination of any discrepancies between professional consensus and public beliefs.

## Methods

### Professional samples

Surveys were posted to all 1710 psychiatrists listed on the Medicare Provider File (Medicare is Australia's national health insurance scheme), a random sample of 2000 GPs listed on the File, all 1628 Australian members of the Australian and New Zealand College of Mental Health Nurses, and a random sample of 2000 psychologists listed in the Victorian Psychologists Registration Board's online database of registered psychologists. Surveys were completed anonymously and separate response cards with identification numbers were used to determine participation or refusal. GPs, psychiatrists and psychologists were sent one reminder letter to encourage participation. Completed surveys were received from 470 GPs, 591 psychiatrists, 522 mental health nurses and 736 psychologists. Response rates were 24.0% (GPs), 35.4% (psychiatrists), 32.3% (mental health nurses) and 40.3% (psychologists).

### Youth and parent samples

The details of these samples have been previously published [14], so are only described briefly here. In 2006, a telephone survey was carried out with a national sample of young Australians aged 12–25 years. If the young person lived at home with a parent, then one parent was randomly invited to be interviewed as well. Interviews were completed for 3746 young people out of 6087 who could be contacted and were confirmed as in scope, giving a response rate of 61.5%. There were 2925 youth respondents with a co-resident parent, of which 2005 completed interviews, giving a response rate of 68.5%.

### Survey questions

The survey was based on a vignette of a young person with a mental disorder [14]. Participants were randomly given a vignette describing a 15 or 21 year old with depression, depression with alcohol misuse, social phobia or psychosis. The vignettes were written to satisfy DSM-IV criteria and were validated by asking the professionals in the current study what was wrong with the person described. (Over 80% of each professional group diagnosed the psychosis vignette with schizophrenia or other psychotic disorder, the depression vignette with a mood disorder and the social phobia vignette with an anxiety disorder. However, diagnosis of comorbid substance-related disorder was lower, ranging from 26% up to 60% depending on the professional group and the age of the person in the vignette [19]). Vignettes were matched to the gender of youth participants, whereas professionals received only male vignettes due to the smaller sample size and the fact that previous research had shown that gender of the vignette had very little effect on responses [20]. After being presented with the vignette, professionals were asked a series of questions to assess their recognition of the disorder in the vignette, beliefs about first aid, interventions, and prevention, and sociodemographic characteristics. The present paper presents data only on the first aid questions, so these are described in detail here. Professionals were asked whether it would be helpful if a friend or family member were to do various first aid actions, youth were asked whether it would be helpful if they provided the first aid to someone they knew and cared about, and parents were asked whether it would be helpful if they provided the first aid to their child. The first aid actions were: 'Listen to his problems in an understanding way. Talk to him firmly about getting his act together. Suggest he seek professional help. Make an appointment for him to see a GP. Ask him whether he is feeling suicidal. Suggest he have a few drinks to forget his troubles. Rally friends to cheer him up. Ignore him until he gets over it. Keep him busy to keep his mind off problems. Encourage him to become more physically active.' Participants could respond 'helpful', 'harmful', 'neither', 'depends' or 'don't know'. These 10 potential first aid strategies were based on previous work on what members of the public report they would do to help someone with a mental disorder [13] and strategies that are described as either helpful or not helpful by a mental health first aid training manual [21].

Previous publications on these data sets have covered young people's and parent's beliefs about help-seeking [22], treatments [18], the effect of substance use on mental disorders [23], mental health first aid strategies [14], stigmatizing attitudes [24], and awareness of a national depression initiative [25]. There have also been publications on clinicians' beliefs about treatments for depression and psychosis [26,27], their recognition of mental disorders in young people [19], and their beliefs about intervention to reduce smoking in people with mental disorders [28].

### Statistical analysis

Responses were dichotomized in two ways: helpful versus all other responses, or harmful versus all other responses. For descriptive purposes, the percent rating each strategy as helpful and as harmful was calculated by vignette and professional group. To examine differences in ratings according to profession and vignette, multiple logistic regressions were carried out predicting helpful or harmful ratings in the combined professional sample. The predictors were profession (with GP as the reference category), type of disorder in the vignette (with depression as the reference category) and age group in the vignette (with 15 years as the reference category). Because of the large sample size and the number of comparisons examined, the 99% CI was used for odds ratios.

Data on the youth and parent samples have been reported previously [14], but are reproduced here to allow comparison with the professional data. The major interest is in how youth and parents rate those first aid strategies about which there is professional consensus on helpfulness or harmfulness. A strategy was classified as recommended by professionals if the mean helpful rating across the four professions and the two vignette ages was >70%. Similarly, a strategy was classified as harmful where the mean harmful rating was >70%. These cutoffs were chosen because they well exceed a majority support for either using or avoiding a first aid strategy. Because of the large sample size of youth and parents, even very small differences in ratings of helpfulness could be statistically significant. Therefore comparisons of public with professionals were made in terms of effect sizes, with medium and large effect sizes noted. Following Rosenthal [29], a medium effect size was defined as a difference in percentages of at least 18 points and a large effect size as a difference of at least 30 points.

### Ethics approval

Approval was given by the University of Melbourne Human Research Ethics Committee.

## Results

Table 1 shows the percentage rating each strategy as likely to be helpful, while Table 2 shows the percentages rating each as likely to be harmful.

**Table 1:** Percentages of professionals, youth and parents believing in the helpfulness of first aid strategies for various disorders

**Group**	**Depression 15 years**	**Depression 21 years**	**Depression with alcohol misuse 15 years**	**Depression with alcohol misuse 21 years**	**Social phobia 15 years**	**Social phobia 21 years**	**Psychosis 15 years**	**Psychosis 21 years**
**Listen to problems in an understanding way**								

GPs	100.0	100.0	100.0	98.3	95.7	100.0	85.4	82.5
Psychiatrists	100.0	95.3	100.0	98.8	100.0	91.8	90.9	88.5
Psychologists	100.0	95.1	98.8	96.7	97.8	95.3	90.8	85.3
Nurses	100.0	97.5	98.6	96.8	100.0	98.5	88.4	91.9
Youth	95.9	96.8	94.1	97.0	95.4	97.0	96.5	96.2
Parents	99.0	97.6	99.9	99.5	98.9	98.8	97.7	94.4

**Talk to firmly about getting act together**								

GPs	2.8	1.5	1.8	1.7	6.5	0.0	0.0	3.5
Psychiatrists	0.0	1.2	4.8	5.9	1.7	0.0	0.0	2.6
Psychologists	0.0	0.0	1.2	0.0	0.0	1.2	0.0	1.1
Nurses	1.3	0.0	1.4	0.0	0.0	0.0	0.0	0.0
Youth	37.0**	29.5*	48.8**	35.6**	28.9*	25.0*	39.4**	30.3*
Parents	22.7*	21.9*	30.9*	27.1*	14.8	18.7*	21.0*	17.9

**Suggest seek professional help**								

GPs	90.3	98.5	86.2	95.0	71.7	82.0	93.8	89.5
Psychiatrists	86.1	91.8	93.5	92.9	84.5	94.5	92.2	94.9
Psychologists	74.0	89.1	77.9	92.3	81.7	88.2	83.9	83.2
Nurses	76.3	84.8	87.8	92.1	71.4	88.2	76.7	83.6
Youth	68.5	75.1	70.3	72.3*	47.9*	64.4*	68.2*	74.8
Parents	73.3	84.4	72.8	85.2	66.3	74.9	77.9	81.1

**Make an appointment for person to see GP**								

GPs	76.4	77.9	87.9	85.0	84.8	69.5	91.7	89.5
Psychiatrists	82.2	82.4	80.6	85.9	77.6	64.4	88.3	87.2
Psychologists	70.8	61.8	64.0	56.0	45.7	47.0	77.0	78.7
Nurses	77.6	75.6	82.4	72.6	50.9	55.9	81.8	80.6
Youth	53.7*	58.4	53.3*	55.7*	39.7*	48.4	50.6*	63.0*
Parents	91.4	88.8	83.2	91.6	77.4	80.1*	87.8	93.5

**Ask whether feeling suicidal**								

GPs	83.3	92.6	82.8	85.0	52.3	63.9	68.8	73.7
Psychiatrists	79.5	78.8	85.5	81.2	56.1	54.2	81.8	84.6
Psychologists	59.4	67.6	61.4	73.3	39.8	44.0	70.1	63.4
Nurses	89.5	78.5	83.8	84.1	54.5	58.2	62.8	80.6
Youth	35.5**	44.4**	38.9**	49.8*	35.9	47.9	40.4**	51.8*
Parents	54.4*	48.7**	52.7*	52.2*	47.0	55.3	54.5	45.4**

**Suggest have few drinks to forget troubles**								

GPs	1.4	0.0	0.0	0.0	0.0	1.6	0.0	0.0
Psychiatrists	0.0	0.0	0.0	0.0	0.0	0.0	0.0	0.0
Psychologists	0.0	0.0	0.0	0.0	1.1	0.0	0.0	1.0
Nurses	0.0	3.8	0.0	1.6	0.0	1.5	0.0	0.0
Youth	5.6	5.4	3.5	3.5	5.4	10.0	4.4	4.2
Parents	0.2	2.3	0.1	2.5	0.0	1.6	0.1	0.7

**Rally friends to cheer up**								

GPs	23.6	30.9	25.9	26.7	11.1	16.7	8.3	12.3
Psychiatrists	20.5	22.4	25.8	25.9	10.3	6.8	14.3	5.2
Psychologists	22.3	20.0	16.3	20.0	12.0	10.7	4.7	4.2
Nurses	17.1	15.2	8.1	11.3	8.9	9.0	0.0	6.5
Youth	74.2**	80.4**	76.8**	80.1**	80.8**	80.1**	75.9**	73.2**
Parents	60.4**	59.0**	54.8**	57.8**	65.4**	64.0**	57.6**	52.7**

**Ignore until gets over it**								

GPs	1.4	0.0	0.0	0.0	2.2	0.0	0.0	3.5
Psychiatrists	4.1	0.0	1.6	2.4	1.7	0.0	0.0	0.0
Psychologists	1.0	0.0	0.0	1.1	0.0	0.0	0.0	2.1
Nurses	0.0	0.0	1.4	0.0	0.0	0.0	0.0	0.0
Youth	2.6	1.1	2.0	1.7	0.6	1.1	1.6	2.0
Parents	0.6	2.6	0.1	0.7	0.2	0.4	0.0	0.4

**Keep busy to keep mind off problems**								

GPs	6.9	13.2	8.6	15.0	15.2	9.8	6.3	7.0
Psychiatrists	5.5	16.7	9.7	7.1	6.9	9.6	5.2	3.9
Psychologists	9.4	9.8	8.1	13.3	9.7	4.7	2.3	4.2
Nurses	7.9	10.3	8.1	12.7	19.6	10.6	4.5	9.7
Youth	67.4**	49.9**	62.0**	55.0**	63.8**	51.7**	68.6**	50.4**
Parents	49.8**	47.3**	58.7**	45.1**	52.1**	46.9**	46.4**	55.5**

**Encourage to become more physically active**								

GPs	75.0	80.9	72.4	78.3	76.1	78.7	39.6	33.3
Psychiatrists	52.1	54.1	61.3	64.7	63.8	57.5	29.9	18.2
Psychologists	71.9	75.4	64.0	82.4	61.3	61.2	32.2	31.6
Nurses	52.6	57.0	64.9	68.3	51.8	61.8	20.5	30.6
Youth	72.8	78.8	78.5	85.8	79.3	83.8*	77.6**	78.8**
Parents	80.9*	81.7	90.1*	88.2	88.7*	88.0*	77.9**	83.9**

*Difference from professionals is at least a medium effect size

**Difference from professionals is at least a large effect size

**Table 2:** Percentages of professionals, youth and parents believing in the harmfulness of first aid strategies for various disorders

**Group**	**Depression 15 years**	**Depression 21 years**	**Depression with alcohol misuse 15 years**	**Depression with alcohol misuse 21 years**	**Social phobia 15 years**	**Social phobia 21 years**	**Psychosis 15 years**	**Psychosis 21 years**
**Listen to problems in an understanding way**								

GPs	0.0	0.0	0.0	0.0	0.0	0.0	0.0	0.0
Psychiatrists	0.0	0.0	0.0	0.0	0.0	0.0	0.0	0.0
Psychologists	0.0	0.0	0.0	0.0	0.0	0.0	0.0	0.0
Nurses	0.0	0.0	0.0	0.0	0.0	0.0	0.0	0.0
Youth	0.8	0.3	0.0	0.0	1.0	0.0	0.8	0.0
Parents	0.0	0.0	0.0	0.0	0.0	0.0	0.4	0.0

**Talk to firmly about getting act together**								

GPs	63.9	57.4	61.4	58.3	69.6	74.6	81.2	77.2
Psychiatrists	80.8	70.6	62.9	47.1	65.5	74.6	71.4	75.6
Psychologists	75.3	74.5	69.0	59.3	80.4	75.9	71.4	67.4
Nurses	89.5	83.5	79.7	66.1	92.7	75.0	81.8	67.7
Youth	34.7**	38.2**	25.5**	36.9**	44.7**	51.8*	33.0**	39.6**
Parents	46.5**	50.3*	44.5*	49.6	66.4	60.5	57.0*	62.2

**Suggest seek professional help**								

GPs	0.0	0.0	0.0	0.0	0.0	0.0	0.0	0.0
Psychiatrists	0.0	0.0	0.0	0.0	1.7	0.0	0.0	0.0
Psychologists	0.0	0.0	0.0	0.0	0.0	0.0	0.0	0.0
Nurses	1.3	0.0	0.0	0.0	0.0	0.0	0.0	1.6
Youth	11.5	7.3	10.4	6.5	21.0*	13.5	12.7	9.1
Parents	7.6	5.0	8.9	5.5	14.8	5.9	6.8	4.9

**Make an appointment for person to see GP**								

GPs	0.0	0.0	0.0	3.3	0.0	0.0	0.0	0.0
Psychiatrists	0.0	0.0	1.6	1.2	3.4	2.7	0.0	0.0
Psychologists	0.0	1.0	1.2	1.1	3.3	4.8	0.0	1.1
Nurses	1.3	2.6	0.0	0.0	1.8	4.4	2.3	1.6
Youth	22.2*	19.7*	23.3*	22.5*	28.8*	32.8*	24.6*	20.5*
Parents	7.6	5.0	8.9	5.6	14.8	5.9	6.8	4.9

**Ask whether feeling suicidal**								

GPs	1.4	1.5	0.0	3.3	4.6	1.6	2.1	3.5
Psychiatrists	0.0	0.0	0.0	0.0	3.5	1.4	1.3	0.0
Psychologists	2.1	2.9	6.0	2.2	8.6	6.0	2.3	2.2
Nurses	0.0	1.3	0.0	1.6	1.8	6.0	0.0	0.0
Youth	34.1**	26.2*	32.7**	22.3*	33.8*	23.2*	35.7**	24.3*
Parents	23.0*	21.5*	21.6*	28.9*	30.6*	23.4*	23.5*	25.0*

**Suggest have few drinks to forget troubles**								

GPs	97.2	97.1	100.0	98.3	97.8	83.6	100.0	98.2
Psychiatrists	94.5	92.9	100.0	100.0	98.3	89.0	96.0	91.0
Psychologists	96.9	97.1	97.7	94.5	95.7	89.3	97.7	92.7
Nurses	100.0	94.9	98.6	96.8	100.0	91.2	95.4	91.9
Youth	85.4	86.6	91.4	91.2	86.0	78.1	87.6	90.2
Parents	97.4	91.6	97.4	96.0	99.3	93.8	98.1	94.2

**Rally friends to cheer up**								

GPs	1.4	7.4	0.0	0.0	20.0	10.0	8.3	15.8
Psychiatrists	2.7	2.4	6.4	3.5	1.7	16.4	20.8	10.4
Psychologists	4.3	7.0	3.5	7.8	15.2	20.2	19.8	13.7
Nurses	11.8	7.6	5.4	6.4	2.9	23.9	2.7	25.8
Youth	6.9	4.9	8.4	5.3	7.6	8.6	10.0	11.3
Parents	8.3	10.8	12.9	10.6	15.4	7.3	19.1	12.1

**Ignore until gets over it**								

GPs	97.2	95.6	98.3	100.0	84.8	85.0	100.0	89.5
Psychiatrists	90.4	96.4	95.2	92.9	87.9	91.8	98.7	98.7
Psychologists	99.0	97.1	96.5	98.9	95.7	95.2	97.7	91.7
Nurses	96.0	100.0	98.6	96.8	98.2	92.6	100.0	100.0
Youth	91.0	94.2	89.9	94.5	94.6	95.0	90.2	93.2
Parents	96.0	95.5	97.0	98.2	95.3	94.0	98.3	96.8

**Keep busy to keep mind off problems**								

GPs	6.9	8.8	12.1	16.7	17.4	11.5	25.0	31.6
Psychiatrists	21.9	17.9	16.1	15.3	24.1	15.1	32.5	23.4
Psychologists	19.8	15.7	20.9	17.8	17.2	18.8	27.6	22.1
Nurses	29.0	12.8	17.6	11.1	19.6	22.7	29.6	32.3
Youth	12.1	25.8	10.1	17.7	10.6	20.8	7.8	23.6
Parents	17.2	19.7	18.4	17.5	19.9	16.8	20.5	14.0

**Encourage to become more physically active**								

GPs	0.0	1.5	0.0	1.7	0.0	1.6	6.2	3.5
Psychiatrists	5.5	0.0	1.6	1.2	0.0	0.0	5.2	5.2
Psychologists	2.1	1.0	0.0	0.0	0.0	1.2	3.4	2.1
Nurses	0.0	3.8	0.0	1.6	0.0	0.0	4.6	4.8
Youth	2.8	2.4	3.9	1.9	1.9	0.8	2.8	2.3
Parents	3.4	4.5	1.3	2.3	1.4	1.6	5.5	1.0

*Difference from professionals is at least a medium effect size

**Difference from professionals is at least a large effect size

### Differences across professions and vignettes

Additional file 1 shows the results when multiple logistic regression was used in the combined professional sample to examine predictors of rating each strategy as likely to be helpful. The additional file also shows the results when examining predictors of rating each strategy as likely to be harmful.

A number of significant differences were found for helpful ratings. *Listen to problems in an understanding way *was recommended less for psychosis than for depression and less for 21-year-olds than 15-year-olds. *Suggest seek professional help *was recommended less by mental health nurses than GPs and more for 21-year-olds than 15-year-olds. *Make an appointment for person to see GP *was recommended more for psychosis than depression, less for social phobia than depression, and less by either psychologists or mental health nurses than GPs. *Ask whether feeling suicidal *was recommended less for social phobia than for depression and less by psychologists than GPs. *Rally friends to cheer up *was generally not seen as a helpful strategy, but was seen as less helpful for psychosis or social phobia than depression, and was recommended less by psychologists and mental health nurses than by GPs. *Keep busy to keep mind off problems *was not generally seen as a helpful strategy, but was recommended less for psychosis than for depression. *Encourage to become more physically active *was recommended less for psychosis than for depression and less by psychiatrists or nurses than GPs.

There were also a number of significant differences for harmful ratings. *Talk to firmly about getting act together *was rated as harmful more by psychologists than GPs, less for depression with alcohol misuse than for depression alone, and for less for 21-year-olds than 15-year olds. *Make an appointment for person to see GP *and *ask whether feeling suicidal *were rated as harmful more for social phobia than for depression. *Suggest have a few drinks to forget troubles *was rated as harmful less for social phobia than depression and less for 21-year-olds than 15-year-olds. *Rally friends to cheer up *was more often rated as harmful by nurses than GPs and more often for psychosis and social phobia than for depression. *Ignore until gets over it *was more often rated as harmful by nurses than by GPs and less often for social phobia than depression. *Keep busy to keep mind off problems *was more often rated as harmful for psychosis than for depression.

### Agreement across professions

While there are many statistically significant differences, it can be seen from the percentages in Tables 1 and 2 that the differences are comparatively small in magnitude and that there is considerable agreement across professions about what are helpful strategies. There were also few differences between what was recommended for adolescents and young adults. Table 3 lists the strategies for which there is substantial agreement that the strategy is likely to be helpful or likely to be harmful. Looking across the disorders in Table 3, it can be seen that there is a lot of overlap in recommendations. The main difference is that social phobia received fewer firm recommendations for helpful strategies.

**Table 3:** Summary of professionals' first aid recommendations

**Recommendation**	**Depression**	**Depression with Alcohol Misuse**	**Social Phobia**	**Psychosis**
Helpful (Mean rating of >70%)	• Listen to problems in an understanding way	• Listen to problems in an understanding way	• Listen to problems in an understanding way	• Listen to problems in an understanding way
	• Suggest seek professional help	• Suggest seek professional help	• Suggest seek professional help	• Suggest seek professional help
	• Make an appointment for person to see GP	• Make an appointment for person to see GP		• Make an appointment for person to see GP
	• Ask whether feeling suicidal	• Ask whether feeling suicidal		• Ask whether feeling suicidal

Harmful (Mean rating of >70%)	• Talk to firmly about getting act together	• Suggest a few drinks to forget troubles	• Talk to firmly about getting act together	• Talk to firmly about getting act together
	• Suggest a few drinks to forget troubles	• Ignore until gets over it	• Suggest a few drinks to forget troubles	• Suggest a few drinks to forget troubles
	• Ignore until gets over it		• Ignore until gets over it	• Ignore until gets over it

### Differences of public beliefs from professional views

The asterisks in Tables 1 and 2 indicate where there were medium or large differences between public and professional ratings. However, some of these differences are with strategies for which there is not professional agreement. The main interest is where there is a difference from the professional beliefs about first aid strategies summarized in Table 3. With strategies recommended by professionals, the major discrepancy is for *ask whether feeling suicidal *which was less frequently rated as helpful and more frequently as harmful by both young people and parents. Young people were also less positive about *make an appointment for person to see GP*, whereas parents were positive about this strategy, consistent with professionals.

There were fewer discrepancies between professional and public views for first aid strategies that were seen as harmful by professionals. However, young people and their parents were less likely to see *talk firmly about getting act together *as harmful.

## Discussion

While there were a number of statistically significant differences between professionals in their beliefs about first aid strategies, these were minor compared to the overall agreement. There were also statistically significant but small differences in beliefs about what strategies would be helpful for different ages and different disorders. More impressive was the degree of agreement about which strategies would be helpful across a range of disorders and for both adolescents and young adults. Based on the professional agreement found here, there are key strategies that members of the public can be advised to carry out that would be helpful across disorders and age groups.

When beliefs of young people and their parents were compared to these professional views, there were several areas of agreement, such as the value of listening to the person's problems in an understanding way, suggesting they seek professional help, not ignoring them, and not suggesting they use substances to cope. A major area of disagreement was in asking about suicidal feelings, which young people and parents were less likely to see as helpful and more likely to see as harmful. This difference may reflect the lay person's fear that mentioning suicide "might put the idea into their head". The beliefs of professionals are supported by the findings of a randomized trial of screening for suicidal thoughts which found no ill effect and some evidence of benefit [30]. The professionals' views support the content of existing training programs like Mental Health First Aid [31] and Applied Suicide Intervention Skills Training (ASIST) [32], which advise the public to talk openly about the subject if they suspect someone is suicidal.

Other areas of discrepancy were found with strategies not endorsed by professionals. Young people and parents were positive about rallying friends to cheer the person up and keeping them busy to keep their mind off problems. However, while professionals did not endorse these strategies, they did not see them as harmful either. Rallying friends could be seen as a type of social support, but it is probably a less effective form of social support than listening in an understanding way. Keeping busy might have some benefits as a distraction technique, but keeping the person's mind off problems is unlikely to promote help-seeking and treatment. Of greater concern is the endorsement by some young people and parents of talking to the person firmly about getting their act together, which was seen as harmful by most professionals. There is evidence from studies of expressed emotion to support the view that criticizing a person with mental illness is not likely to be helpful [33].

This study has a number of limitations. The first aid strategies investigated here are only a small subset of the possibilities and should not be seen as exhaustive. There may be important areas that were not covered by the survey questions. Another limitation is that only health professionals were used as a source of expertise, whereas consumers and carers may have much expertise to offer in the area of first aid strategies. An alternative approach which overcomes these limitations is to carry out consensus studies of what are appropriate first aid strategies using panels of consumers and carers, as well as clinicians. A number of such studies have now been carried out [34-37], looking for first aid strategies that all groups rate as likely to be helpful. The findings of these studies are consistent with the present results, supporting listening to the person, asking about suicidal feelings, encouraging professional help-seeking, and avoiding negative interactions. Finally, there could be unknown sample biases associated with the low response rate for the professional samples. The response rate was lower than in similar surveys a decade ago [20,38] and may reflect the declining response rates of health professionals to postal surveys in Australia and elsewhere [39], or the focus of the survey on the youth age group which some clinicians may not deal with in their practice. However, the major interest was in finding strategies about which there was high agreement across professional groups, rather than in precisely estimating the beliefs of each profession from a representative sample. Non-response from clinicians with less interest in youth mental health may actually lead to better quality of opinion, even if less representative.

## Conclusion

These findings could be used to guide programs to educate the public about what first aid actions are appropriate. Some basic messages could be promoted universally through information campaigns. More detailed messages would require individual training programs like Mental Health First Aid. For some first aid strategies (e.g. asking about suicidal feelings), there is a need to promote greater public knowledge about appropriate actions. However, for strategies where there is already substantial agreement between the public and professionals (e.g. listening in an understanding way), the need may be to train specific skills rather than to increase knowledge. The existing training programs have been aimed at adults who want to provide first aid to others, including youth, rather than at increasing the first aid skills of young people. Given that young people nominate peers as an important source of potential help for mental health problems [11,18], some consideration needs to be given to how to provide them with some basic first aid skills. Although adolescents may not be mature enough to provide optimal first aid, the reality is that their peers are already turning to them as a source of help, so some guidance is necessary. If the present findings were used to guide the content of interventions, it is important that those interventions be evaluated in controlled trials to assess the benefits of the first aid strategies taught.

## Competing interests

The authors declare that they have no competing interests.

## Authors' contributions

AFJ designed the study, analysed the data and had the major role in writing the paper. AJM co-designed and ran the professional surveys and contributed to writing the paper. AW co-designed the public and professional surveys and edited the paper.

## Pre-publication history

The pre-publication history for this paper can be accessed here:



## Supplementary Material

Additional file 1 Odds ratios from multiple logistic regression analyses predicting helpful ratings and harmful ratings for each first aid strategy. Click here for file
