# Supplementary material for: First aid strategies that are helpful to young people developing a mental disorder: beliefs of health professionals compared to young people and parents
Source: BMC Psychiatry. 2008 Jun 8;8:42. doi: 10.1186/1471-244X-8-42 (PMC2438354; doi:10.1186/1471-244X-8-42)
Supplement: Additional file 1 — Odds ratios from multiple logistic regression analyses predicting helpful ratings and harmful ratings for each first aid strategy. [file 1471-244X-8-42-S1.doc]

Odds ratios from multiple logistic regression analyses predicting helpful ratings for each first aid strategy

| **Predictor** | **OR** | **99% CI** |
| --- | --- | --- |
| **Listen to problems in an understanding way** | | |
| Psychiatrist | 1.01 | 0.45-2.24 |
| Psychologist | 0.87 | 0.41-1.86 |
| Nurse | 1.36 | 0.56-3.36 |
| GP (reference group) | 1.00 |  |
| Psychosis vignette | 0.13 | 0.06-0.30 |
| Social phobia vignette | 0.61 | 0.22-1.72 |
| Depression & alcohol misuse vignette | 1.10 | 0.34-3.52 |
| Depression vignette (reference group) | 1.00 |  |
| Age of vignette (21 years) | 0.52 | 0.30-0.92 |
| **Talk to firmly about getting act together** | | |
| Psychiatrist | 0.95 | 0.31-2.91 |
| Psychologist | 0.19 | 0.04-1.05 |
| Nurse | 0.17 | 0.02-1.29 |
| GP (reference group) | 1.00 |  |
| Psychosis vignette | 1.16 | 0.23-6.01 |
| Social phobia vignette | 1.26 | 0.24-6.50 |
| Depression & alcohol misuse vignette | 2.77 | 0.69-11.05 |
| Depression vignette (reference group) | 1.00 |  |
| Age of vignette (21 years) | 0.94 | 0.35-2.58 |
| **Suggest seek professional help** | | |
| Psychiatrist | 1.33 | 0.77-2.28 |
| Psychologist | 0.65 | 0.41-1.03 |
| Nurse | 0.61 | 0.37-0.99 |
| GP (reference group) | 1.00 |  |
| Psychosis vignette | 1.09 | 0.70-1.70 |
| Social phobia vignette | 0.83 | 0.54-1.27 |
| Depression & alcohol misuse vignette | 1.39 | 0.88-2.20 |
| Depression vignette (reference group) | 1.00 |  |
| Age of vignette (21 years) | 1.84 | 1.33-2.55 |
| **Make an appointment for person to see GP** | | |
| Psychiatrist | 0.93 | 0.61-1.41 |
| Psychologist | 0.35 | 0.24-0.51 |
| Nurse | 0.57 | 0.38-0.85 |
| GP (reference group) | 1.00 |  |
| Psychosis vignette | 1.78 | 1.21-2.61 |
| Social phobia vignette | 0.49 | 0.35-0.69 |
| Depression & alcohol misuse vignette | 1.04 | 0.74-1.47 |
| Depression vignette (reference group) | 1.00 |  |
| Age of vignette (21 years) | 0.85 | 0.66-1.09 |
| **Ask whether feeling suicidal** | | |
| Psychiatrist | 0.95 | 0.65-1.40 |
| Psychologist | 0.45 | 0.32-0.64 |
| Nurse | 0.93 | 0.63-1.39 |
| GP (reference group) | 1.00 |  |
| Psychosis vignette | 0.82 | 0.58-1.17 |
| Social phobia vignette | 0.31 | 0.22-0.43 |
| Depression & alcohol misuse vignette | 1.08 | 0.75-1.56 |
| Depression vignette (reference group) | 1.00 |  |
| Age of vignette (21 years) | 1.15 | 0.90-1.47 |
| **Suggest have few drinks to forget troubles** | | |
| Psychiatrist | 0.00 |  |
| Psychologist | 0.66 | 0.05-8.78 |
| Nurse | 2.26 | 0.26-19.70 |
| GP (reference group) | 1.00 |  |
| Psychosis vignette | 0.31 | 0.02-5.60 |
| Social phobia vignette | 0.89 | 0.12-6.44 |
| Depression & alcohol misuse vignette | 0.29 | 0.02-5.21 |
| Depression vignette (reference group) | 1.00 |  |
| Age of vignette (21 years) | 3.30 | 0.41-26.20 |
| **Rally friends to cheer up** | | |
| Psychiatrist | 0.81 | 0.53-1.23 |
| Psychologist | 0.65 | 0.43-0.98 |
| Nurse | 0.43 | 0.27-0.70 |
| GP (reference group) | 1.00 |  |
| Psychosis vignette | 0.27 | 0.16-0.44 |
| Social phobia vignette | 0.44 | 0.28-0.68 |
| Depression & alcohol misuse vignette | 0.91 | 0.63-1.31 |
| Depression vignette (reference group) | 1.00 |  |
| Age of vignette (21 years) | 1.05 | 0.77-1.42 |
| **Ignore until gets over it** | | |
| Psychiatrist | 1.41 | 0.28-7.18 |
| Psychologist | 0.64 | 0.10-4.01 |
| Nurse | 0.22 | 0.01-4.00 |
| GP (reference group) | 1.00 |  |
| Psychosis vignette | 0.90 | 0.16-5.14 |
| Social phobia vignette | 0.49 | 0.16-4.22 |
| Depression & alcohol misuse vignette | 1.12 | 0.22-5.79 |
| Depression vignette (reference group) | 1.00 |  |
| Age of vignette (21 years) | 0.70 | 0.19-2.57 |
| **Keep busy to keep mind off problem** | | |
| Psychiatrist | 0.79 | 0.46-1.38 |
| Psychologist | 0.75 | 0.44-1.28 |
| Nurse | 1.01 | 0.59-1.74 |
| GP (reference group) | 1.00 |  |
| Psychosis vignette | 0.49 | 0.27-0.90 |
| Social phobia vignette | 1.03 | 0.62-1.69 |
| Depression & alcohol misuse vignette | 1.02 | 0.63-1.67 |
| Depression vignette (reference group) | 1.00 |  |
| Age of vignette (21 years) | 1.22 | 0.84-1.79 |
| **Encourage to become more physically active** | | |
| Psychiatrist | 0.45 | 0.32-0.64 |
| Psychologist | 0.72 | 0.51-1.01 |
| Nurse | 0.47 | 0.33-0.68 |
| GP (reference group) | 1.00 |  |
| Psychosis vignette | 0.22 | 0.16-0.30 |
| Social phobia vignette | 0.93 | 0.68-1.27 |
| Depression & alcohol misuse vignette | 1.25 | 0.91-1.72 |
| Depression vignette (reference group) | 1.00 |  |
| Age of vignette (21 years) | 1.14 | 0.91-1.44 |

Odds ratios from multiple logistic regression analyses predicting harmful ratings for each first aid strategy

| **Predictor** | **OR** | **99% CI** |
| --- | --- | --- |
| **Listen to problems in an understanding way** | | |
| Could not be computed because of zero frequencies | | |
| **Talk to firmly about getting act together** | | |
| Psychiatrist | 1.07 | 0.75-1.50 |
| Psychologist | 1.23 | 0.88-1.72 |
| Nurse | 1.93 | 1.32-2.83 |
| GP (reference group) | 1.00 |  |
| Psychosis vignette | 0.95 | 0.67-1.34 |
| Social phobia vignette | 1.10 | 0.77-1.56 |
| Depression & alcohol misuse vignette | 0.57 | 0.41-0.78 |
| Depression vignette (reference group) | 1.00 |  |
| Age of vignette (21 years) | 0.74 | 0.58-0.95 |
| **Suggest seek professional help** | | |
| Could not be computed because of zero frequencies | | |
| **Make an appointment for person to see GP** | | |
| Psychiatrist | 2.40 | 0.29-19.87 |
| Psychologist | 3.58 | 0.49-26.22 |
| Nurse | 4.05 | 0.54-30.70 |
| GP (reference group) | 1.00 |  |
| Psychosis vignette | 0.88 | 0.12-6.37 |
| Social phobia vignette | 4.60 | 1.07-19.80 |
| Depression & alcohol misuse vignette | 1.69 | 0.32-9.00 |
| Depression vignette (reference group) | 1.00 |  |
| Age of vignette (21 years) | 1.68 | 0.60-4.70 |
| **Ask whether feeling suicidal** | | |
| Psychiatrist | 0.31 | 0.07-1.45 |
| Psychologist | 1.86 | 0.71-4.86 |
| Nurse | 0.61 | 0.17-2.22 |
| GP (reference group) | 1.00 |  |
| Psychosis vignette | 1.19 | 0.32-4.36 |
| Social phobia vignette | 3.73 | 1.28-10.85 |
| Depression & alcohol misuse vignette | 1.44 | 0.42-4.96 |
| Depression vignette (reference group) | 1.00 |  |
| Age of vignette (21 years) | 0.94 | 0.45-1.98 |
| **Suggest have few drinks to forget troubles** | | |
| Psychiatrist | 0.73 | 0.32-1.64 |
| Psychologist | 0.74 | 0.34-1.62 |
| Nurse | 0.94 | 0.39-2.24 |
| GP (reference group) | 1.00 |  |
| Psychosis vignette | 0.77 | 0.36-1.61 |
| Social phobia vignette | 0.50 | 0.25-0.99 |
| Depression & alcohol misuse vignette | 2.00 | 0.77-5.19 |
| Depression vignette (reference group) | 1.00 |  |
| Age of vignette (21 years) | 0.34 | 0.19-0.63 |
| **Rally friends to cheer up** | | |
| Psychiatrist | 1.32 | 0.73-2.39 |
| Psychologist | 1.56 | 0.89-2.72 |
| Nurse | 2.56 | 1.46-4.51 |
| GP (reference group) | 1.00 |  |
| Psychosis vignette |  |  |
| Social phobia vignette | 3.72 | 2.18-6.34 |
| Depression & alcohol misuse vignette | 3.96 | 2.33-6.72 |
| Depression vignette (reference group) | 1.00 |  |
| Age of vignette (21 years) | 0.92 | 0.65-1.32 |
| **Ignore until gets over it** | | |
| Psychiatrist | 1.04 | 0.52-2.05 |
| Psychologist | 1.76 | 0.85-3.63 |
| Nurse | 2.76 | 1.11-6.84 |
| GP (reference group) | 1.00 |  |
| Psychosis vignette | 1.06 | 0.46-2.44 |
| Social phobia vignette | 0.40 | 0.20-0.80 |
| Depression & alcohol misuse vignette | 1.16 | 0.50-2.71 |
| Depression vignette (reference group) | 1.00 |  |
| Age of vignette (21 years) | 0.84 | 0.49-1.43 |
| **Keep busy to keep mind off problem** | | |
| Psychiatrist | 1.39 | 0.91-2.13 |
| Psychologist | 1.33 | 0.88-1.99 |
| Nurse | 1.51 | 0.98-2.32 |
| GP (reference group) | 1.00 |  |
| Psychosis vignette | 1.90 | 1.31-2.74 |
| Social phobia vignette | 1.10 | 0.74-1.64 |
| Depression & alcohol misuse vignette | 0.96 | 0.64-1.43 |
| Depression vignette (reference group) | 1.00 |  |
| Age of vignette (21 years) | 0.82 | 0.63-1.08 |
| **Encourage to become more physically active** | | |
| Psychiatrist | 1.33 | 0.42-4.24 |
| Psychologist | 0.69 | 0.19-2.46 |
| Nurse | 1.06 | 0.30-3.78 |
| GP (reference group) | 1.00 |  |
| Psychosis vignette | 2.56 | 0.98-6.67 |
| Social phobia vignette | 0.22 | 0.03-1.59 |
| Depression & alcohol misuse vignette | 0.40 | 0.09-1.83 |
| Depression vignette (reference group) | 1.00 |  |
| Age of vignette (21 years) | 1.00 | 0.44-2.28 |
